# Non-*Aspergillus* molds

**DOI:** 10.1016/j.jhlto.2025.100382

**Published:** 2025-08-25

**Authors:** Emily M. Eichenberger, Maria Alejandra Mendoza, John W. Baddley

**Affiliations:** a Emory University School of Medicine, Atlanta, Georgia; b Division of Infectious Diseases, Department of Internal Medicine, University of Utah, Salt Lake City, Utah; c Johns Hopkins University School of Medicine, Baltimore, Maryland

**Keywords:** *Mucor*, *Fusarium*, *Scedosporium*, *Lomentospora*, invasive fungal infection, lung transplant, heart transplant

## Abstract

Non-*Aspergillus* molds, including Mucorales, *Scedosporium, Lomentospora,* and *Fusarium* species, are a significant cause of morbidity and mortality in heart and lung transplant recipients. These organisms have a marked propensity for angioinvasion leading to thrombosis and tissue infarction and disseminated infection. General risk factors for infection with these non-*Aspergillus* molds include older age, augmented immunosuppression (e.g., hypogammaglobulinemia, neutropenia, T-cell depletion), presence of endobronchial stent, and airway ischemia. Infection is uncommon, but in many cases may ensue following respiratory colonization, particularly in lung transplant recipients. Timing of infection varies, although many invasive fungal infections occur within the first year following transplantation. Diagnosis is challenging and often delayed. Imaging is recommended to localize infection and to guide sampling of infected tissue for culture and histopathology. Management of these rare molds in lung and heart transplant recipients presents a major therapeutic challenge due to intrinsic resistance patterns, delayed diagnosis, and the complex pharmacologic interactions in this population. In general, lipid preparations of amphotericin B or azole antifungals (voriconazole, posaconazole, isavuconazole) are frequently used for treatment. Investigational therapies such as fosmanogepix or olorofim are promising as future treatment modalities for some of these difficult-to-treat non-*Aspergillus* molds*.*

## Background

Non-*Aspergillus* molds are multidrug-resistant, emerging pathogens, and are a significant cause of morbidity and mortality in heart and lung transplant recipients.^1^, ^2^ The major non-*Aspergillus* molds that cause infection among transplant recipients include Mucorales (i.e., *Mucor*, *Rhizopus*), *Scedosporium, Lomentospora,* and *Fusarium* species. These organisms have a marked propensity for angioinvasion leading to thrombosis, tissue infarction, and disseminated infection.^3^, ^4^ General risk factors for infection with these non-*Aspergillus* molds include older age, augmented immunosuppression (e.g., hypogammaglobulinemia, neutropenia, T-cell depletion), presence of an endobronchial stent, and airway ischemia.^5^

**Figure 1:**
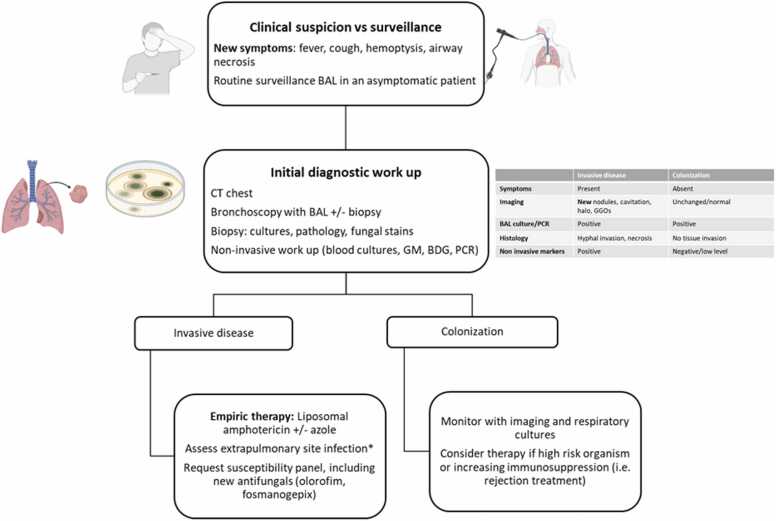
Proposed clinical decision tree. BAL, bronchoalveolar lavage; BDG, beta-d-glucan; CT, computed tomography; GGO, ground glass opacities; GM, galactomannan; PCR, polymerase chain reaction. *Brain, sinus, skin, gastrointestinal tract, and imaging recommended if these symptoms are present.

The timing of invasive fungal infections varies, though most occur within the first year after transplantation.^5^ While non-*Aspergillus* invasive fungal infections is uncommon, it often follows colonization of the respiratory tract. Distinguishing colonization from true infection is essential. In the absence of accompanying symptoms or compatible radiographic changes, the presence of fungi from a nonsterile site (e.g., bronchoalveolar lavage fluid) is considered colonization rather than infection (Figure 1).^1^, ^6^ The management of colonized patients is not standardized, but in many cases, experts recommend initiating antifungal therapy.

Management of these rare molds in lung and heart transplant recipients presents a major therapeutic challenge due to intrinsic resistance patterns, delayed diagnosis, and the complex pharmacologic interactions in this population. The currently recommended antifungal therapies may be challenging to use. For example, azole antifungals are potent CYP3A4 inhibitors and significantly increase levels of tacrolimus, cyclosporine, and sirolimus, necessitating frequent dose adjustments and monitoring. Amphotericin B formulations may exacerbate nephrotoxicity when combined with calcineurin inhibitors. Novel investigational agents such as fosmanogepix or olorofim may offer treatment advantages in the future. Herein, we review the epidemiology, clinical presentation, diagnosis, and management of several important non-*Aspergillus* molds, including Mucorales, *Scedosporium, Lomentospora,* and *Fusarium*, in heart and lung transplant recipients. Discussion of the rarer non-*Aspergillus* molds such as *Paecilomyces*, *Purpureocillium,* and *Rasamsonia* is not covered in this review.

## Mucorales

### Epidemiology and pathogenesis

Mucorales are found in soil and decaying organic material. Infection may be acquired via inhalation of aerosolized spores, direct inoculation of skin from trauma, ingestion, or, rarely, through donor-derived transmission.^7^ The incidence of Mucorales infection in heart and lung transplant recipients is approximately 8 and 14 per 1,000 patients, respectively.^8^ Infections have been observed from the immediate postoperative period up to 11.5 years post-transplant, with reports of 41% of cases occurring within the first month and 78% within the first year.^1^ Additional risk factors for mucormycosis in heart and lung recipients include comorbid diabetes mellitus and renal failure.^1^

### Clinical manifestations

Infections due to Mucorales, referred to as mucormycosis, frequently manifest as pulmonary or rhino-orbital-cerebral disease, but may also involve other anatomical sites such as the skin or gastrointestinal tract.^9^ Among lung transplant recipients, the most common site of infection is the lung, accounting for more than 60% of cases, followed by disseminated and rhinocerebral disease^1^, ^8^, ^10^ The most commonly reported symptoms of pulmonary mucormycosis included fever (54.8%), followed by cough (29%), dyspnea (19.4%), chest pain (12.9%), and hemoptysis (9.7%).^11^ Patients with rhinocerebral mucormycosis may present with sinus pain, facial swelling, fever, and toothache.^8^ Gastrointestinal mucormycosis in heart and lung transplant recipients is uncommon, but may present as abdominal pain, gastrointestinal bleeding, and fever.^8^, ^12^, ^13^

Radiographically, pulmonary mucormycosis may present with multiple pulmonary nodules or pleural effusions (Table 1). Presence of a reverse halo sign, an area of ground glass opacity surrounded by a consolidative ring, or vessel occlusion on computed tomography (CT) pulmonary angiography, should raise suspicion for mucormycosis over *Aspergillus* infection.^14^, ^15^ However, clinicians should note that this finding is not specific to Mucorales. A reverse halo sign can be seen in other infections, including paracoccidioidomycosis, mycobacterial infections, as well as in systemic inflammatory and neoplastic diseases.^16^

**Table 1:** Diagnosis of Non-*Aspergillus* Molds

	Culture	Histology	Radiologic findings
Mucorales	Rapidly growing cotton candy–like colonies. Tissue culture may be falsely negative in up to 50% cases.^14^, ^15^	Wide (diameter 6-25 µm) ribbon-like, nonseptate or pauci-septate hyphae branching at 45°-90° angles.^17^	Lung: Pleural effusion; reverse halo sign; vessel occlusion on pulmonary angiogram.^14^, ^15^ Sinus: Sinus opacification and mucosal edema are indistinguishable from bacterial sinusitis. Boney erosion is a late finding.^15^, ^18^ Orbits: Edema or thickening of the orbital muscles^18^, ^19^
*Lomentospora prolificans*	Colonies appear black. Semiselective culture media such as Sce-Sel+ media or inhibitory mold agar and brain heart infusion agar often helpful. Growth is inhibited by cycloheximide.^20^	Pigmented septate hyphae with flask-shaped and annellated conidiogeneous cells. May see intravascular conidiation or conidiation within tissue.^20^	Lung: Diffuse or nodular infiltrates with or without cavitation, consolidation, pleural effusion may be present.^21^, ^22^ Brain: Enhancing brain lesions, meningeal enhancement.^22^
*Scedosporium* species	*Scedosporium apiospermum* and *Scedosporium boydii* typically form cottony gray-white colonies *Scedosporium aurantiacum* forms yellow-gray colonies that darken to gray-brown over time. Semiselective culture media such as Sce-Sel+ media or inhibitory mold agar and brain heart infusion agar often helpful.^20^	Irregularly acute branching (60°-70° angles) septate, hyaline hyphae. Branching may occasionally form an “H”-shaped pattern. May see intravascular conidiation or conidiation within tissue.^20^	Lungs: Variable presentation; single to multiple nodules, with or without cavitation, often without crescent formation. Can also present with focal infiltrates, lobar infiltrates, bilateral diffuse infiltrates, or as necrotizing pneumonia.^21^, ^22^, ^23^ Brain: Ring-enhancing lesions on CT, or hyperintense lesions on MRI^24^, ^25^, ^26^
*Fusarium* species	Blood cultures positive in 40% of invasive cases. Colonies can be velvet or cotton-like in texture, and present in a variety of colors (e.g., pink, red, white, yellow, or gray).^4^	Hyaline septate filaments with acute angle branching and banana-shaped macroconidia. Adventitious sporulation may be present.^4^	Lungs: Micronodules without a halo sign, centrilobular micronodules, or ground glass infiltrates.^27^ Sinuses: CT may demonstrate mucosal thickening and opacification.^28^

Abbreviations: CT, computed tomography; MRI, magnetic resonace imaging.

Rhino-orbital-cerebral involvement commonly involves the maxillary and ethmoid sinuses, and patients may present with fever, localized facial pain and swelling, nasal discharge, and cranial nerve deficits.^8^ Imaging of the sinuses may demonstrate mucosal thickening and sinus opacification. The absence of bony erosion on imaging does not exclude mucormycosis as a potential diagnosis, as osseous destruction is typically a late finding and not always present. Magnetic resonace imaging (MRI) may reveal vascular occlusion, thrombosis, and infarction due to the predilection for invasion of the blood vessel walls.^15^, ^18^

Bronchial anastomotic infections are uncommon but have been reported.^29^ Clinical manifestations may include changes in spirometry, noisy breathing, or difficulty clearing secretions. Suspicion for anastomotic infection may arise from imaging findings such as airway irregularities or extraluminal air, or from bronchoscopic evidence of pseudomembrane formation.^30^

### Diagnosis

Diagnosis of mucormycosis is challenging and often delayed (Table 1). Imaging is recommended for all difficult-to-diagnose mold infections to localize infection and guide sampling of infected tissue for culture and histopathology.^28^ In general, CT is the preferred initial imaging modality for lung and sinuses. CT is rapid, widely available, and can be useful to guide diagnostic procedures. CT may also be used for imaging the abdomen and bone when involvement is suspected.^28^ Use of intravenous (IV) contrast enhancement CT can be helpful to detect angioinvasion.^31^ MRI is more sensitive than CT for identification of early sinus involvement, orbital, and intracranial spread of infection, and is recommended when sinus disease is suspected.^31^ MRI is also the modality of choice for evaluating central nervous system (CNS) involvement given its superiority in detecting early parenchymal changes, infarcts, and perineural spread.^32^ Limitations of MRI included long scan times and contraindications in patients with certain implantable devices. Fluorodeoxyglucose-positron emission tomography is an alternative imaging modality when MRI is unavailable or contraindicated, and can be an adjunct in determining the extent of infection.^33^

From tissue samples, Mucorales typically grow within 3 to 7 days, appearing as cotton candy–like colonies on most fungal culture media (e.g., Sabouraud agar and potato dextrose agar) incubated at 25°C to 30°C.^14^, ^15^ On direct microscopy, Mucorales appear as wide (diameter 6-25 μm) ribbon-like, nonseptate, or pauci-septate hyphae with branching at 45° to 90° angles. The use of fluorescent brighteners such as Calcofluor White can help identify fungal elements on direct microscopy.^17^ Up to half of all Mucorales infections fail to grow in culture despite being observed on histopathology because the nonseptate hyphae are extremely fragile and can be damaged during tissue manipulation.^15^, ^34^, ^35^ To optimize culture yield, it is recommended to slice tissue rather than grind specimens.^15^ Notification of the microbiology and pathology labs ahead of specimen acquisition may improve organism recovery.

Fungal biomarkers, including 1,3-β-D-glucan (BDG) and *Aspergillus* galactomannan, are typically negative in patients with mucormycosis, as the cell wall of Mucorales contains little to no BDG, and does not contain galactomannan. Importantly, a positive BDG or *Aspergillus* galactomannan does not automatically rule out a Mucorales infection, as a mixed infection may be present.^15^

Molecular diagnostic methods are frequently used to aid in species-level identification.^28^, ^36^ Matrix-assisted laser desorption/ionization–time-of-flight mass spectrometry (MALDI-TOF) is increasingly used for ulture specimens.^15^ A serum quantitative polymerase chain reaction (PCR) test has been developed for Mucormycosis, which holds promise for earlier detection of disease, with reported sensitivity and specificity of 85.2% and 89.8%, respectively.^37^ PCR testing for Mucorales in bronchoalveolar lavage fluid is also available and highly specific, but has a low positive predictive value for infection.^38^ A positive bronchoalveolar lavage PCR cannot distinguish between colonization and infection, and should be interpreted with caution, particularly in the lung transplant population that may be prone to airway colonization.

### Management

**Figure 2:**
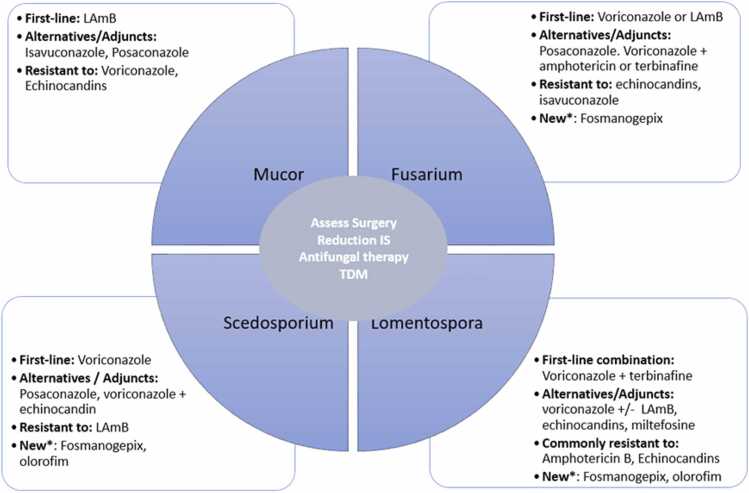
Antifungal treatment options for non-*Aspergillus* mold infections. IS, immunosuppression; LAmB, liposomal amphotericin B; TDM, therapeutic drug monitoring. *Under clinical trials, they should be considered as second-line therapies.

A combination of surgery and antifungal therapy is preferred for Mucorales infections (Table 2; Figure 2). Surgical debridement is recommended for purposes of disease control and for obtaining diagnostic specimens, and should accompany antifungal therapy when feasible.^15^, ^39^ Liposomal amphotericin B (LAmB) is recommended as first-line therapy in solid organ transplant recipients with recommended 5 to 10 mg/kg/day depending on the severity of infection, with a dose of 10 mg/kg/day in cases of CNS involvement.^15^ Early initiation of therapy is essential.^40^, ^41^

**Table 2:** Management of Non-*Aspergillus* Molds

	First line	Alternative/salvage
Mucorales	LAmB 5-10 mg/kg/day^a^ + surgical debridement	Isavuconazole or posaconazole alone or in combination with LAmB
*Lomentospora prolificans*	Voriconazole + terbinafine + surgical debridement	Voriconazole alone or in combination with LAmB, an echinocandin, or miltefosine
*Scedosporium* species	Voriconazole + surgical debridement	Posaconazole or voriconazole + echinocandin
*Fusarium* species	LAmB 5 mg/kg/day or ABLC or voriconazole	Voriconazole in combination with terbinafine or LAMB, or Posaconazole

Abbreviations: ABLC, amphotericin B lipid complex; CNS, central nervous system; IV, intravenous; LAmB, liposomal amphotericin B.

For all infections above, the following doses are recommended:

Voriconazole: 6 mg/kg IV or PO twice daily for 2 doses followed by 4 mg/kg IV or PO twice daily thereafter.

Isavuconazole: 200 mg IV or PO 3 times daily for 6 doses, followed by 200 mg daily thereafter.

Posaconazole: 300 mg PO tablet or IV twice daily for 2 doses followed by 300 mg PO or IV daily thereafter. Posaconazole tablet is preferred to suspension.

Terbinafine: 250 mg PO twice daily.

Echinocandin: micafungin 150 mg IV daily; caspofungin 70 mg IV on day 1 followed by 50 mg or 70 mg IV daily thereafter, depending on severity of infection; anidulafungin 200 mg IV on day 1 followed by 100 mg IV daily thereafter; may consider increasing anidulafungin dose by 50%-75% in critically ill or obese patients.

Therapeutic drug monitoring is recommended for azole therapies.

a CNS involvement requires LAmB dosed at 10 mg/kg/day.

Isavuconazole, available as IV or oral formulations, is commonly used as step-down or salvage therapy or in patients with pre-existing renal disease, due to its bioavailability, tolerability, and efficacy.^42^ In the VITAL open-label study, isavuconazole demonstrated comparable efficacy to amphotericin B, although only 3 solid organ transplant (SOT) patients were included.^43^ Data on its use specifically in thoracic transplant recipients remain limited.^44^, ^45^ A post hoc analysis of VITAL reported 63.6% survival at day 84 for patients with CNS mucormycosis,^46^ and preclinical models have shown that isavuconazole penetrates the blood-brain barrier.^47^

Posaconazole, particularly in its delayed-release tablet and IV formulations, is another alternative agent, often used in outpatient or salvage settings. Posaconazole is also generally considered an alternative second-line azole for those unable to tolerate amphotericin B.^48^ Voriconazole lacks activity against Mucorales and is not recommended for treatment.^49^ Therapeutic drug monitoring of azoles should be performed to ensure therapeutic concentrations.

Treatment duration spans several weeks to months and is ultimately individualized based on radiologic and symptomatic improvement, source control, as well as the patient’s degree of immune suppression.

### Prognosis

Despite antifungal treatment, mucormycosis continues to carry a high mortality rate, ranging from 32% to 62%^1^, ^50^ in lung transplant recipients and 50%^8^ in heart recipients. Mortality approaches 100% in disseminated mucormycosis.^8^, ^51^ Coinfection with other fungi, malnutrition, delay in diagnosis, and lack of surgical intervention are all associated with worse outcomes.^1^, ^51^, ^52^

### Key points

• Infections due to Mucorales fungi frequently manifest as pulmonary or rhino-orbital-cerebral disease.
• Up to half of all Mucorales fail to grow on culture despite being observed on histopathology because the nonseptate hyphae are extremely fragile and can be damaged during tissue manipulation.
• A combination of surgery and antifungal therapy is preferred for Mucorales infections.

## Scedosporium and lomentospora

### Epidemiology and pathogenesis

*Scedosporium* species and *Lomentospora prolificans* are responsible for approximately 1% of all fungal infections in SOT recipients, with lung recipients being the most frequently affected.^53^ Lung transplant recipients are also more likely to be colonized pre- or post-transplant owing to underlying lung disease (e.g., cystic fibrosis, chronic obstructive pulmonary disease).^20^ Notably, one study identified that invasive infection with *Scedosporium* or *L. prolificans* was preceded by colonization in 36% of cases.^54^ It is currently a matter of debate as to whether colonization with *Scedosporium* species or *L. prolificans* is a contraindication to lung transplant.^55^, ^56^, ^57^

These organisms are found in soil, manure, and wastewater. Acquisition of infection mainly occurs via inhalation of conidia or inoculation of fungi from the environment in the setting of trauma.^20^ Donor-derived infections have also been documented, notably in cases wherein the organs are transplanted from donors with near-drowning.^20^, ^58^, ^59^

### Clinical and radiologic manifestations

*Scedosporium* and *L. prolificans* infections often originate in the lungs, though dissemination is common, complicating approximately 47% of cases in solid organ transplant recipients. Disseminated infection most commonly involves the lungs, skin, endovascular system, and CNS, while sinus involvement is rare.^20^, ^60^, ^61^ Mediastinitis has also been reported in thoracic transplant recipients.^54^ Fungemia is frequently observed in *L. prolificans* and *S. boydii* infections, but is uncommon in *S. apiospermum* infections.^20^

Radiographic pulmonary findings in both *Scedosporium* and *L. prolificans* infections are variable. CT of the lungs may demonstrate solitary or multiple pulmonary nodules with or without cavitation, or bilateral diffuse infiltrates, occasionally with an accompanying pleural effusion.^21^, ^22^, ^23^ Necrotizing pneumonia may also be seen.^23^ Patients with CNS involvement will typically have ring-enhancing lesions on CT or meningeal enhancement.^24^, ^25^, ^26^, ^62^

### Diagnosis

*Scedosporium* species and *L. prolificans* can be cultured on standard mycological media; however, growth from nonsterile sites may be suppressed by overgrowth of competing fungi such as *Aspergillus* or *Candida* (Table 1).^63^ Semiselective culture media such as Sce-Sel+ media, inhibitory mold agar, and brain heart infusion agar can be helpful.^28^, ^64^, ^65^ Microscopically, *L. prolificans* presents as pigmented septate hyphae with flask-shaped and annellated conidiogeneous cells.^28^ Intravascular conidiation or conidiation within tissues may be seen and is specifically what facilitates its dissemination. *Scedosporium* species are irregularly acute branching (60°-70°) septate, hyaline hyphae that can be difficult to distinguish from *Aspergillus.*^20^ Branching can occasionally be seen bridging 2 parallel hyphae to form an “H”-shaped pattern.^63^, ^66^ Intravascular and intratissue conidiation may also be identified.^20^

Species-level identification of *L. prolificans* and *Scedosporium* is made by morphological identification or internal transcribed spacer sequencing. MALDI-TOF can reliably identify *L. prolificans* at the species level^67^ and *Scedosporium* at the genus level.^28^, ^36^ There are currently no commercial serological or standardized PCR assays for the diagnosis of *Scedosporium* or *L. prolificans*.

### Management

*Scedosporium* and *L. prolificans* infections are some of the most therapeutically challenging mold infections (Table 2; Figure 2). Antifungal susceptibility patterns differ substantially between species: *Scedosporium* species often show variable susceptibility to azole antifungals, while *L. prolificans* is characteristically pan-resistant to most available antifungal agents. Due to their relative rarity, no randomized controlled trials have evaluated the efficacy of specific antifungal regimens for scedosporiosis or lomentosporiosis. For both infections, surgical debridement is recommended when feasible.^28^

Voriconazole is the preferred first-line agent for *Scedosporium* infections. Salvage regimens may include combination therapy with voriconazole and an echinocandin, or posaconazole monotherapy; however, posaconazole is significantly less effective against *Scedosporium* relative to voriconazole.^28^, ^20^ LAmB monotherapy is not recommended.^28^ Isavuconazole is not active against *Scedosporium* species.^68^ As with all invasive fungal infections, therapeutic drug monitoring of azoles is critical to ensure therapeutic concentrations.

*L. prolificans* is among the most resistant molds encountered in transplant recipients. It exhibits high minimum inhibitory concentrations (MICs) to nearly all available antifungal agents, including amphotericin B, triazoles, and echinocandins.^69^ While *L. prolificans* is resistant to voriconazole in vitro*,* combination therapy using a voriconazole backbone is recommended.^28^ Guidelines specifically recommend a combination of voriconazole with terbinafine as a first-line treatment strategy,^28^, ^33^ citing evidence from in vitro synergy studies and clinical case series.^20^, ^69^ Alternative and salvage regimens for *Lomentospora* infections may include voriconazole alone or in combination with amphotericin B, an echinocandin, or miltefosine.^28^, ^33^

Inhalational and localized antifungal therapy have also been used in selected cases of lomentosporiosis, particularly for pulmonary infections. This includes nebulized or inhaled formulations of amphotericin B lipid complex, LAmB, and voriconazole,^70^ though clinical efficacy remains anecdotal and is not guideline-endorsed. Treatment duration for *Lomentospora* and *Scedosporium* infections is not defined and contingent upon improvement of underlying symptoms and signs, but typically requires a minimum of 3 months.

### Prognosis

*Scedosporium* infections are associated with high mortality rates, estimated at approximately 57% in heart and 55% in lung transplant recipients.^71^ *L. prolificans* infections carry an even worse prognosis, with mortality reaching up to 75%^72^ in heart and approaching 100% in lung.^61^ However, available outcomes data remain limited. In general, extrapulmonary or disseminated infections, as well as early post-transplant infection, are linked to significantly increased risk of mortality in both *Scedosporium* and *Lomentospora* infections.^54^^,^^73^

### Key points

• Invasive infection with *Scedosporium or L. prolificans* is often preceded by colonization, especially in lung transplant recipients.
• *Scedosporium* species often show variable susceptibility to azole antifungals, while *L. prolificans* are characteristically pan-resistant to available antifungal agents.
• Voriconazole is the preferred first-line agent for *Scedosporium* infections.
• Voriconazole in combination with terbinafine is a recommended treatment regimen for *L. prolificans.*

## Fusarium

### Epidemiology and pathogenesis

*Fusarium* species are ubiquitous in the environment, found in the soil, water (including seawater and community water systems), and in the air.^4^ Infection may be acquired via inhalation or entry through broken skin.^2^ Donor-derived fusariosis has been reported, but is rare.^64^ *Fusarium* infections in heart and lung recipients are fortunately infrequent.^74^, ^75^, ^76^, ^77^

### Clinical and radiological manifestations

*Fusarium* infections are notorious for their frequent hematogenous dissemination, often presenting with persistent fevers, positive blood cultures, and painful erythematous nodular skin lesions with central necrosis. In lung transplant recipients, respiratory symptoms are the most commonly reported clinical manifestation.^77^ Imaging of the lungs often reveals micronodules without a halo sign, centrilobular micronodules, or ground glass infiltrates.^27^

### Diagnosis

Fungemia is a common manifestation of fusariosis (Table 1). *Fusarium* species grow easily on different media without cycloheximide.^4^ Microscopically, *Fusarium* has banana-shaped macroconidia,^4^ and in tissue, they appear as septate hyaline filaments with acute angle branching, similar to *Aspergillus*. Identification at the species level requires molecular methods or MALDI-TOF.^78^ Patients with fusariosis may have a positive serum galactomannan or a positive 1,3 beta-D-glucan.^79^ When available, molecular-based testing may be helpful for species identification.^28^

### Management

*Fusarium* infections are particularly difficult to treat due to intrinsic antifungal resistance and frequent hematogenous dissemination in immunocompromised hosts (Table 2; Figure 2). A lipid formulation of amphotericin B or voriconazole is the recommended first-line treatment for *Fusarium* infection,^28^, ^33^ though voriconazole activity may vary depending on the species and isolate.^80^, ^81^ Combination therapy with amphotericin B and voriconazole, or voriconazole and terbinafine, may also be considered due to species-dependent variability in in vitro susceptibility profiles, or to overcome difficulties in achieving therapeutic voriconazole levels in the setting of high MICs.^28^, ^33^ Terbinafine has shown in vitro activity against *Fusarium verticillioides* . Combination therapy using terbinafine with azoles, especially voriconazole, has demonstrated synergistic effects in several in vitro studies.^82^, ^83^, ^84^ Of note, clinical evidence supporting voriconazole plus terbinafine combination therapy is limited to case reports.^28^, ^33^

Posaconazole demonstrates in vitro activity against some *Fusarium* isolates and has been employed as salvage therapy or when first-line agents are contraindicated.^85^ Isavuconazole has demonstrated variable in vitro activity, with MICs often ≥16 µg/ml, suggesting limited efficacy against *Fusarium* species.^86^, ^87^ Echinocandins are not recommended as they are not effective against *Fusarium* species.^88^

### Prognosis

Mortality data for thoracic organ transplant recipients with *Fusarium* infection are limited. However, 2 case series report mortality rates in lung transplant recipients ranging from 33% to 83%.^77^, ^89^ Severe and persistent neutropenia and disseminated infection are risk factors for mortality.^77^

### Key points

• *Fusarium* infections often present with persistent fevers, positive blood cultures, and painful erythematous nodular skin lesions with central necrosis.
• *Fusarium* infections are particularly difficult to treat due to intrinsic antifungal resistance.
• A lipid formulation of amphotericin B, or voriconazole, is the recommended first-line treatment for *Fusarium* infection.
• Combination therapy with amphotericin B and voriconazole, or voriconazole and terbinafine, may also be considered.

## Prevention

Antifungal prophylaxis or pre-emptive therapy is a critical aspect of infection prevention in thoracic organ transplantation. Lung transplant recipients in particular are at high risk for invasive fungal infections due to factors such as airway manipulation, immunosuppression, colonization, ischemic airway complications, and continuous contact of the graft with air. Heart transplant recipients face a lower but notable risk, particularly when supported by extracorporeal membrane oxygenation, ventricular assist devices, or with prolonged intensive care unit stays.^90^ In cases where a thoracic transplant recipient or donor has a pathogenic non-*Aspergillus* mold, including *Scedosporium, L. prolificans*, or *Mucor*, isolated, a thorough evaluation for colonization vs infection should be performed, with use of clinical, radiological, and histological findings (Figure 1). The transplant community remains in equipoise as to whether colonization warrants targeted antifungal prophylaxis.^54^, ^55^, ^91^, ^92^, ^93^, ^94^ If infection is identified, treatment should be offered as outlined above and in Table 2. Counseling of the thoracic transplant recipient on avoiding activities that place them at increased risk for acquiring invasive fungal infections, such as home composting, contact with sewage/decaying material, visiting construction sites, and manipulating heating/air cooling vents and filters.^39^

## Future directions

An important issue with Non-*Aspergillus* molds is the associated high mortality, often related to delay in diagnosis and ineffective therapeutics due to intrinsic organism resistance. The development of novel antifungal agents offers hope for managing invasive fungal infections caused by resistant molds such as Mucorales, *Scedosporium, Lomentospora*, and *Fusarium* species, particularly in transplant recipients where therapeutic options remain limited. There are several active compounds in advanced development.

### Fosmanogepix

Fosmanogepix (APX001), a prodrug of manogepix, targets the fungal enzyme Gwt1, which is involved in glycosylphosphatidylinositol-anchor biosynthesis. It has demonstrated broad-spectrum activity, including against yeasts, dimorphic fungi, *Scedosporium*, and some *Fusarium* species.^95^, ^96^ Notably, it possesses high oral bioavailability and is currently undergoing evaluation in a Phase 3 clinical trial investigating the safety and efficacy of fosmanogepix in adult patients with invasive mold infections caused by *L. prolificans,* Mucorales, *Fusarium* species, *Aspergillus* species, and other multidrug-resistant molds (ClinicalTrials.gov NCT06925321). The trial is enrolling 2 cohorts of patients: those undergoing primary therapy for the invasive mold infection (patients will receive study drug or standard of care), and those undergoing salvage therapy (patients all receive study drug). The primary outcome is all-cause mortality at day 42, with key secondary outcomes including overall response of treatment success at day 42, 84, and 180 days, and all-cause mortality at day 42, 84, and 180 days.

### Olorofim

Olorofim is the first-in-class agent of the orotomide family, targeting the fungal enzyme dihydroorotate dehydrogenase, which is critical for pyrimidine biosynthesis. It exhibits broad-spectrum activity against hyaline molds, dimorphic fungi, *Scedosporium* species, and *L. prolificans*.^97^ Olorofim is not active against Mucorales, as Mucorales lack the class 2 dihydroorotate dehydrogenase enzyme.^98^ A Phase IIb clinical trial was recently published demonstrating efficacy and good tolerability of olorofim in patients with invasive fungal disease with limited to no treatment options, including patients with *Scedosporium* (N = 22), *L. prolificans* (N = 26), and *Fusarium* (N = 3).^99^

## Conclusions

Non-*Aspergillus* molds, including Mucorales, *Scedosporium, Lomentospora,* and *Fusarium* species, represent a significant challenge in the care of heart and lung transplant recipients. These infections are associated with high morbidity and mortality due to their angioinvasive nature, diagnostic complexity, and intrinsic antifungal resistance. The distinction between colonization and infection is critical, particularly in lung transplant recipients in whom respiratory colonization is more common and may precede infection. Early identification of infection remains difficult and is often delayed due to nonspecific clinical presentation and limitations in current diagnostic modalities. Management of these infections is complicated by the limited efficacy of available antifungal agents and their significant pharmacologic interactions with immunosuppressive therapies. Investigational agents such as fosmanogepix and olorofim will hopefully lead to improved management in the future.

## Author contributions

**Emily M. Eichenberger:** Conceptualization, Writing (original draft), Writing (editing and reviewing), Visualization. **Maria Alejandra Mendoza:** Conceptualization, Writing (original draft), Writing (editing and reviewing), Visualization. **John W. Baddley:** Conceptualization, Writing (editing and reviewing), Visualization, Project administration, Supervision.

## Disclosure statement

John Baddley reports a relationship with Elion that includes consulting or advisory. John Baddley reports a relationship with Pulmocide that includes consulting or advisory. The other authors declare that they have no known competing financial interests or personal relationships that could have appeared to influence the work reported in this paper.
